# Spatial segregation of home ranges between neighbouring colonies in a diurnal raptor

**DOI:** 10.1038/s41598-018-29933-2

**Published:** 2018-08-06

**Authors:** Jacopo G. Cecere, Salvatore Bondì, Stefano Podofillini, Simona Imperio, Matteo Griggio, Egidio Fulco, Andrea Curcio, Delphine Ménard, Ugo Mellone, Nicola Saino, Lorenzo Serra, Maurizio Sarà, Diego Rubolini

**Affiliations:** 1 Area Avifauna Migratrice, Istituto Superiore per la Protezione e la Ricerca Ambientale (ISPRA), via Ca’ Fornacetta 9, I-40064 Ozzano Emilia, (BO) Italy; 2 Laboratorio di Zoogeografia ed Ecologia Animale (LABZEA), Dipartimento STEBICEF, Università degli Studi di Palermo, Via Archirafi 18, I-90123 Palermo, Italy; 3 Dipartimento di Scienze e Politiche Ambientali, Università degli Studi di Milano, via Celoria 26, I-20133 Milano, Italy; 4 Dipartimento di Biologia, Università degli Studi di Padova, via U. Bassi 58/B, I-35131 Padova, Italy; 5 Studio Naturalistico Milvus, via F.lli Perito snc, I-85010 Pignola, (PZ) Italy; 6 Vertebrates Zoology Research Group, Departamento de Ciencias Ambientales y Recursos Naturales, University of Alicante, Apdo. 99, Alicante, E-03080 Spain

## Abstract

Enhancement of information transfer has been proposed as a key driver of the evolution of coloniality. Transfer of information on location of food resources implies that individuals from the same colony share foraging areas and that each colony can be associated to a specific foraging area. In colonial breeding vertebrates, colony-specific foraging areas are often spatially segregated, mitigating intercolony intraspecific competition. By means of simultaneous GPS tracking of lesser kestrels (*Falco naumanni*) from neighbouring colonies, we showed a clear segregation of space use between individuals from different colonies. Foraging birds from different neighbouring colonies had home ranges that were significantly more segregated in space than expected by chance. This was the case both between large and between small neighbouring colonies. To our knowledge, the lesser kestrel is the only terrestrial species where evidence of spatial segregation of home ranges between conspecifics from neighbouring colonies has been demonstrated. The observed spatial segregation pattern is consistent with the occurrence of public information transfer about foraging areas and with the avoidance of overexploited areas located between neighbouring colonies. Our findings support the idea that spatial segregation of exploited areas may be widespread among colonial avian taxa, irrespective of colony size.

## Introduction

Coloniality occurs when conspecifics gather in groups to reproduce close to one another and exploit shared resources, often showing reduced territoriality^1^. The ecological factors promoting the evolution of colonial breeding have long been puzzling to evolutionary biologists^2^. Evans *et al*.^3^ argued that enhancing information use is the main advantage of colonial breeding and that it may have contributed more than any other factor to the evolution of coloniality. The transfer of information may allow individuals to gather experience during high-energy demanding life-cycle stages, such as reproduction, reducing costly trials and errors. In a colony environment, there are indeed several kinds of information that can be either intentionally or inadvertently shared among colony members^3^. The “public information” that can be exploited by conspecifics may be disparate, including for example cues useful for sexual choice^4^, nesting habitat selection^5^, foraging^6,7^ and anti-predator defence^8–10^.

In habitats where food resource distribution is both spatially and temporally predictable, individuals - particularly in long-lived species - can rely on memory and cognitive maps for targeting food^11^. In the case of patchy, ephemeral and unpredictable food resources, the use of cognitive maps may not be sufficient for efficiently targeting food and individuals can greatly benefit from the recent experience of conspecifics^12,13^. Transfer of social information about the location of profitable foraging areas may occur at the colony site, where individuals can actively (as proposed by the “Information Centre Hypothesis”^6^) or inadvertently^14,15^ share information on foraging locations. Moreover, social information may be shared outside the colony site, which may occur by means of so-called “local enhancement” processes occurring at the foraging grounds, whereby individuals searching for food are attracted to feeding aggregations of other individuals^16^.

Most of the studies on foraging areas exploited by colonial central-place foragers concerns marine top-predators, especially pinnipeds^17^ and seabirds (e.g. albatrosses, shearwaters, cormorants and gannets), the vast majority of which (>90%) breed colonially^18^. Individuals from different colonies often show colony-specific and well-defined foraging areas, which do not overlap with those belonging to neighbouring conspecific colonies^19–24^. As argued by the “diplomacy” hypothesis^20^, spatial segregation of foraging individuals from different colonies may mitigate intraspecific competition for resources between conspecifics breeding in different colonies. By foraging in spatially segregated areas, conspecifics from different colonies may thus “diplomatically” avoid interference competition for food resources^20^. For instance, it has been shown that inter-colony competition could be one of the main factors driving the at-sea distribution of pelagic foraging birds^25^. Both local enhancement and the transfer of information at the colony site have been hypothesized to be the most important mechanisms generating and maintaining specific foraging areas exploited by individuals belonging to the same colony^23^. Such a spatial arrangement pattern of foraging areas is peculiar of colonial species (*sensu* Danchin *et al*.^1^) and does not occur, for instance, in eusocial insects, where space partitioning between nests originates and is maintained by aggressive interactions^26–28^. In the latter case, spatial arrangement of neighbouring nests often results from the destruction of the newer nest by killing or ejecting founding queens^27^.

During the breeding period, colonial species are central-place foragers (*sensu* Orians & Pearson^29^), with individuals foraging outside the colony, sometimes very far from the breeding site (e.g. seabirds^30^, seals^31^ and bats^32^), and consistently returning to the colony (the “central place”) to perform parental duties. The progressive depletion of foraging areas around the breeding sites leads individuals to both increase foraging ranges (the “Ashmole’s halo” effect^33^) and, in the case of neighbouring colonies, to avoid moving towards adjacent colonies when searching for food. This, in turn, may generate and/or reinforce spatial segregation of foraging areas among individuals from neighbouring colonies^23^.

We analyzed the spatial distribution of home ranges of individuals of a landbird species breeding in neighbouring colonies. We focused on the lesser kestrel (*Falco naumanni*), a small (ca. 120 g) diurnal colonial raptor. The lesser kestrel mainly nests in holes and crevices of anthropogenic structures (roofs, ancient monuments, buildings) and forages in farmland habitats surrounding breeding sites^34^, where it targets invertebrates and small vertebrates (mice, lizards)^35,36^. In such farmland landscapes, lesser kestrel prey can be patchily distributed, highly ephemeral and unpredictable during the species’ breeding season, since habitat characteristics change continuously as a function of seasonal processes (changes in primary productivity affecting prey distribution/availability) and agricultural practices (including pesticide applications, harvesting, stubble burning, ploughing)^37^. Hence, information gathered during previous years or during the pre-breeding period might not be sufficient to identify profitable foraging areas, leading us to hypothesize that lesser kestrels should exploit social information to target profitable hunting grounds. This is corroborated by the observation that lesser kestrels, similarly to other colonial raptors such as vultures (e.g. family *Aegypiinae*) and the Eleonora’s falcon (*Falco eleonorae*), commonly forage in groups, both during the breeding and the non-breeding season^35^.

By analysing GPS information collected during the entire nestling-rearing stage (ca. 30 days) from simultaneously tracked individuals, we investigated the occurrence of spatial segregation between birds from neighbouring colonies in two geographically distinct lesser kestrel populations (Apulia and Sicily, both in Southern Italy). According to the “diplomacy” hypothesis, we predicted spatial segregation of home ranges (assessed by means of the utilization distribution^38^) between lesser kestrels from neighbouring colonies to occur because: 1) resources are expected to be depleted in the surroundings of colony sites (Ashmole’s halo)^39^ and 2) lesser kestrels forage in groups, suggesting that they are highly likely to rely on social information to target ephemeral productive foraging areas.

## Results

Individual home range size largely differed between the two geographical populations (Table 1), being ca. one order of magnitude larger among Apulian compared to Sicilian birds [95% Kernel Density Estimation (95% KDE), Apulia: 138.8 km^2^ (84.5 s.d., n = 18 individuals); Sicily: 13.0 km^2^ (59 s.d., n = 6 individuals); Mann-Whitney U test, Z = 3.6, *p* < 0.001]. No significant sex differences in home range size emerged among Apulian birds [95% KDE, males: 121.7 km^2^ (73.7 s.d., n = 12); females: 172.8 km^2^ (101.1 s.d., n = 6); Mann-Whitney U test, Z = 0.84, *p* = 0.40)], while sex differences could not be tested for Sicilian birds because a single male was tracked.

**Table 1:** Summary information of GPS tracking data for each individual included in the study.

Bird identity	Study area	Colony	Sex	N GPS positions	Hatching date	End tracking date	Days tracked	95% KDE area (km^2^)
H207147	Apulia	GRA	F	245	161	191	22	266.34
H207149	Apulia	GRA	M	927	170	200	29	205.29
H207151	Apulia	GRA	M	803	167	197	28	62.88
H207154	Apulia	GRA	M	1195	165	195	26	69.94
H207155	Apulia	GRA	F	424	165	188	19	109.77
H207156	Apulia	GRA	F	987	171	198	26	206.08
H207200	Apulia	GRA	F	409	168	198	24	48.35
H207204	Apulia	GRA	M	993	168	198	24	307.05
H208463	Apulia	GRA	M	514	171	194	20	149.75
H207172	Apulia	ALT	M	681	175	199	23	79.86
H207174	Apulia	ALT	M	295	173	187	13	122.4
H207175	Apulia	ALT	F	733	169	199	29	303.13
H207176	Apulia	ALT	M	1137	178	208	29	90.97
H207180	Apulia	ALT	M	1253	172	202	29	67.98
H207181	Apulia	ALT	F	363	160	190	20	103.38
H207216	Apulia	ALT	M	436	173	203	28	133.66
H207220	Apulia	ALT	M	148	177	188	10	45.51
H207222	Apulia	ALT	M	1192	172	202	27	125.25
T58200	Sicily	TOR	F	281	135	152	16	11.19
T67675	Sicily	TOR	F	879	135	165	29	11.78
TK7884	Sicily	CAN	F	515	140	170	29	18.58
TK7885	Sicily	CAN	M	1055	137	167	29	4.09
T69718	Sicily	SAN	F	651	142	172	29	11.59
TK7978	Sicily	SAN	F	804	148	177	28	20.54

In Apulia, data were collected during the 2016 breeding season, whereas in Sicily data were collected during the 2015 breeding season. Within a given study area, birds from different colonies were tracked simultaneously (see also Methods). Colony size was ca. 1000 pairs for both Gravina in Puglia (GRA) and Altamura (ALT), 16 for Torrevecchia (TOR), 11 for Canalotto (CAN) and 32 for San Gregorio (SAN); sex: M = male; F = female; dates are expressed in days since January 1.

**Figure 1:**
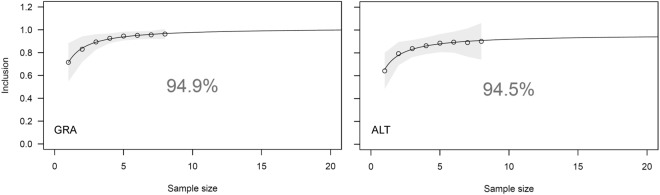
Results of the representativeness analysis showing that the sample of tracked individuals reliably represents the variability in space use of birds from each Apulian colony (GRA: Gravina in Puglia, n = 9 individuals; ALT: Altamura, n = 9 individuals). Circles indicate the average proportion of out-of-sample GPS positions located within the 95% KDE areas estimated from sampled positions (Inclusion) for 100 random draws of sample sizes, from 1 to 8 individuals. Grey bars indicate variability of inclusion value for 100 random draws of tracked individuals, and the solid line represents the fitted nonlinear regression line. Inclusion rate (and thus representativeness of the tracking dataset) is based on the estimated asymptote of the nonlinear regression^40^.

**Figure 2:**
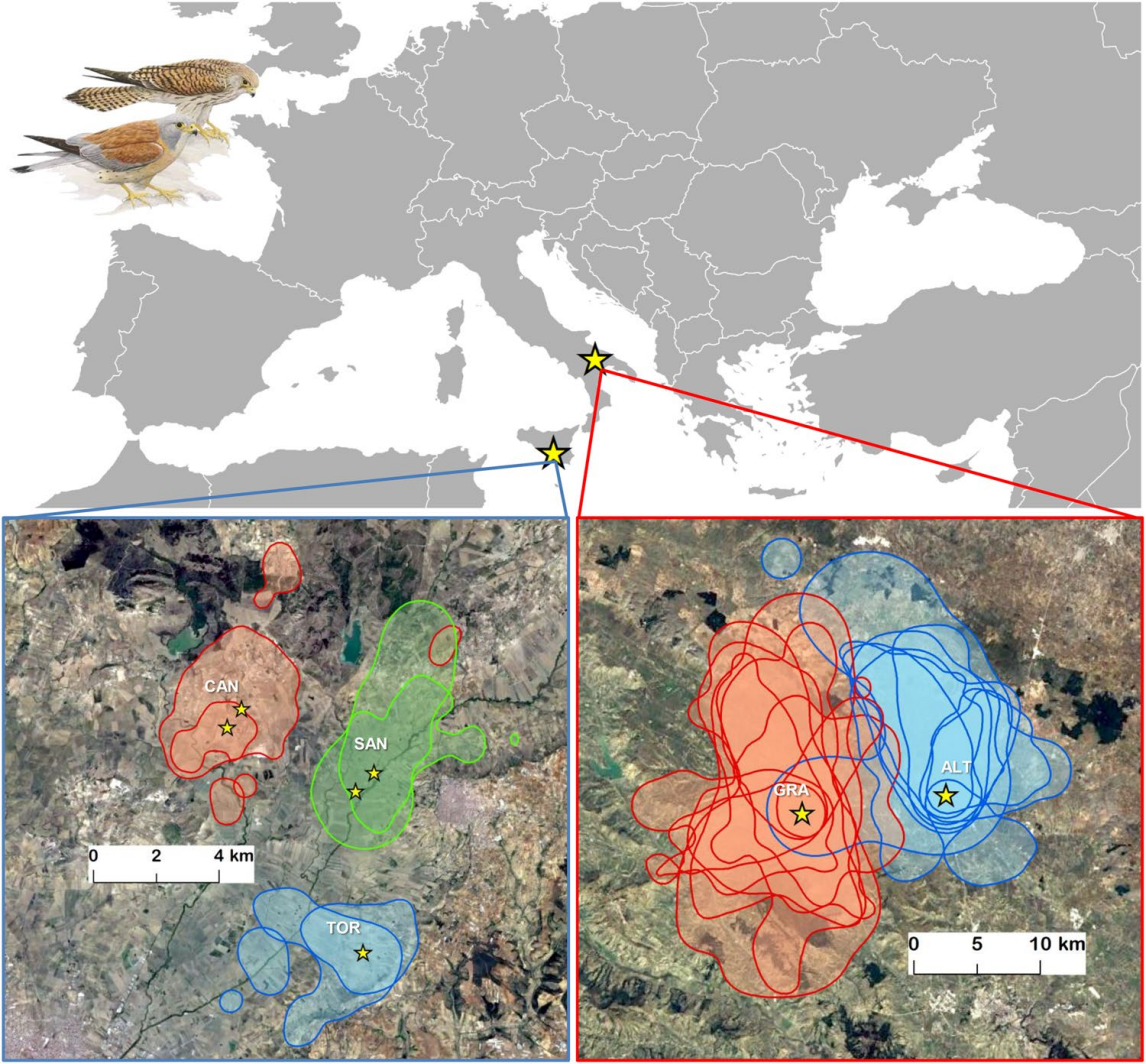
Home ranges (95% KDE) of lesser kestrels breeding at three colony sites in Sicily (bottom left; n = 6 individuals) and at two colony sites in Apulia (bottom right; n = 18 birds). Within each panel, home ranges of the same colour denote birds from the same colony (GRA = Gravina in Puglia, ALT = Altamura, CAN = Canalotto, SAN = Sangregorio, TOR = Torrevecchia) and breeding sites of tracked birds are marked with yellow stars. Satellite images were downloaded from Google EarthPro ver. 7.3.0.3832 3832 (sources: “Gravina in Puglia and Altamura”, coordinates 40.82°N - 16.39°E, 12 March 2016–14 August 2017, Map data © 2018 Google; “Gela”, coordinates 37.14°N - 14.31°E, 12 March 2016–14 August 2017; Map data © 2018 TerraMetrics) and elaborated with ArcGIS ver. 10.2.1 for Desktop. Lesser kestrel drawing is by U. Catalano and has the ISPRA copyright.

Representativeness of tracked individuals from the two Apulian colonies, Gravina in Puglia and Altamura, was very high (95% KDE: >94%; Fig. 1), indicating that we captured most of the variability in space use by individuals from the target colonies. Moreover, the steep curves of the representativeness analysis (Fig. 1) indicated that birds belonging to same colony showed highly overlapping home ranges (see also Fig. 2). Although we could not test for representativeness of tracked individuals from the Sicilian colonies due to the small sample size, Fig. 1 suggests that even a few individuals can well represent the space use of the target colony (e.g. with 2 individuals representativeness is >75%, a relatively high value^40^.

**Figure 3:**
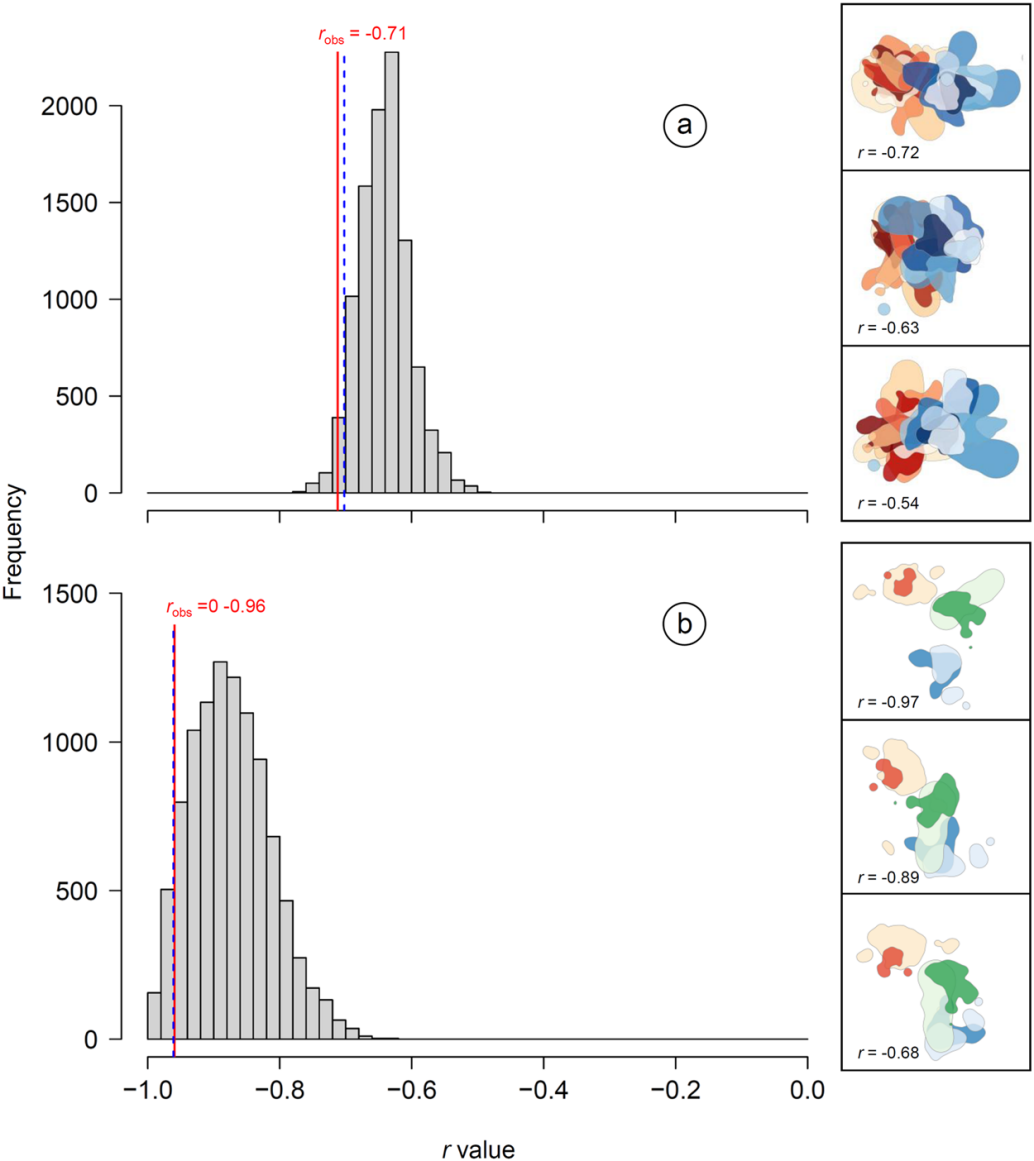
Frequency distribution of randomized *r* values obtained from random rotations of home ranges (with breeding site as the anchor point) in a) Apulia and b) Sicily. *r* values were computed by correlating the matrix of Utilization Distribution Overlap Index (UDOI) values with the matrix of colony membership (0 = individuals belonged to the same colony; 1 = individuals belonged to different colonies) (see Methods for details). More negative *r* values denote greater spatial segregation of home ranges between lesser kestrels from neighbouring colonies (see Fig. 2). The observed *r* value (*r*_obs_), resulting from the spatial distribution of home ranges shown in Fig. 2, is highlighted with a (continuous) red line within each panel. The 95% empirical quantile of the frequency distribution of randomized *r* values is shown with a (broken) blue line. Representative examples of random rotations of home ranges (and the corresponding *r* value) for each study population are shown on the right insets of each panel (home ranges of birds from different colonies are depicted with colour shadings corresponding to those used in Fig. 2); for simplicity, overlapping home ranges are represented with 95% KDEs.

Individual home ranges of birds from the two different Apulian colonies showed a very limited overlap (Fig. 2). The overlap between home ranges of individuals from neighbouring colonies, computed by means of the Utilization Distribution Overlap Index (UDOI)^41,42^, was very low, varying between 0 and 0.11 (mean value = 0.01). At the same time, the UDOI between individuals from the same colony varied between 0.03 and 1.53 (mean value = 0.53). By randomly rotating individual home ranges, we showed that birds from the two Apulian colonies had home ranges that were significantly more spatially segregated than expected by chance according to UDOI values (*r*_obs_ = −0.71, *p*_rand_ = 0.024; Fig. 3). The pattern for the Sicilian colonies, albeit based on a very small sample size, was even more striking (*r*_obs_ = −0.96, *p*_rand_ = 0.057; Fig. 3): UDOI values between individual home ranges of birds belonging to the three neighbouring colonies were indeed 0 or close to 0 in all comparisons (a single comparison had a value of 0.0002), while those between birds from the same colony varied between 0.63 and 1.17 (mean value = 0.89). For Apulia, results of the randomization procedure for assessing home range segregation were strengthened when considering only GPS positions located outside the urban area of the cities where lesser kestrels breed (see Methods) (*p*_rand_ = 0.004).

**Figure 4:**
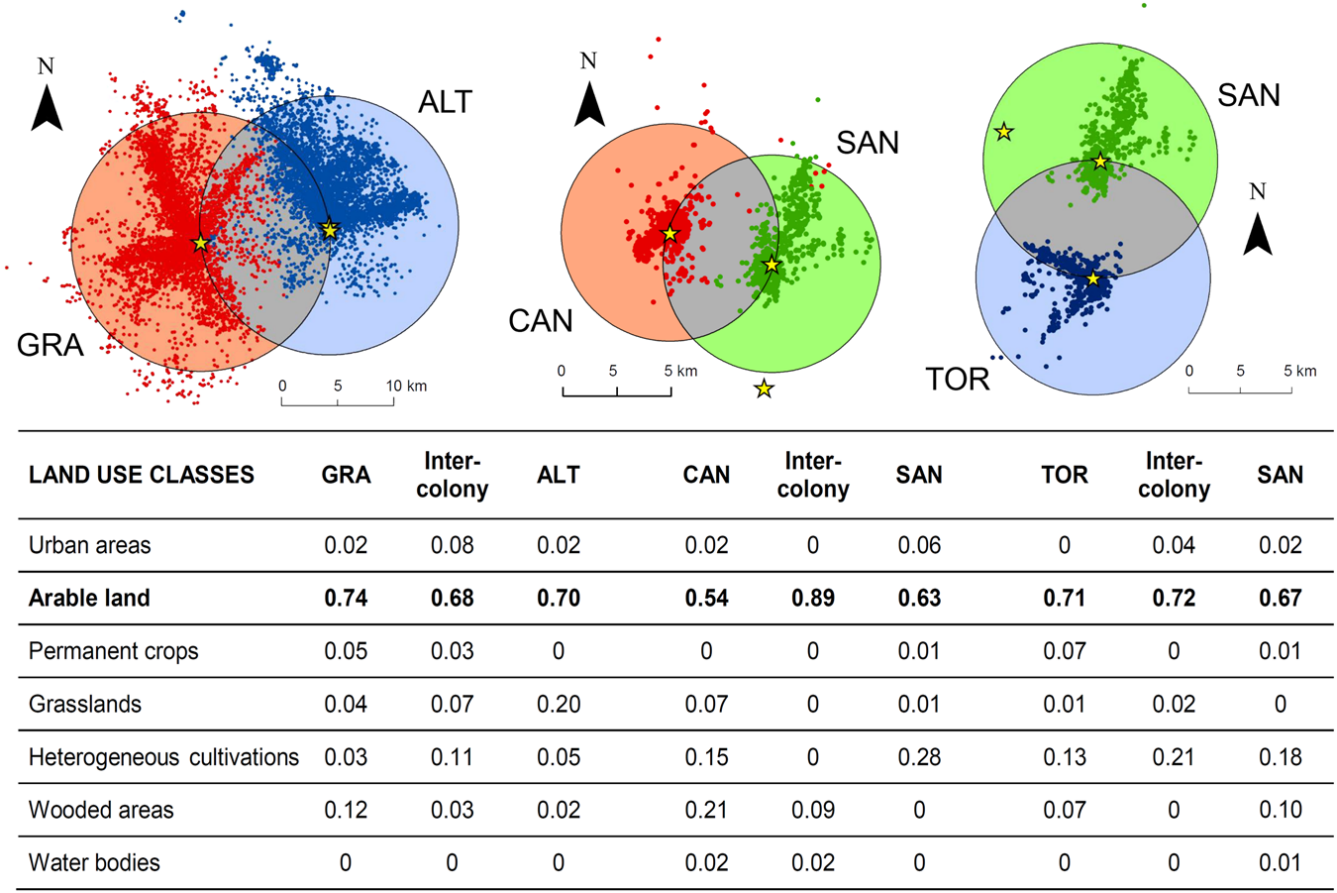
Proportion of land use classes within outer- (orange, blue or green areas) and inter-colonies areas (grey areas). Points represent all recorded GPS positions for a given colony (different colours for neighbouring colonies) and stars represent breeding sites of GPS-tagged birds in each colony (GRA = Gravina in Puglia, ALT = Altamura, CAN = Canalotto, SAN = Sangregorio, TOR = Torrevecchia). The radius of each buffer around colonies (centered on the mean of breeding sites positions) was calculated as the distance between the two neighbouring colonies. Land use classes were identified by means of ArcGIS 10.2.1 for Desktop according to the Corine Land Cover 2012 classification. Arable land (in bold) represents the main foraging habitat for the lesser kestrel in the study areas.

We could rule out that the observed patterns of spatial segregation resulted from the presence of unsuitable foraging habitats in the inter-colony areas. Indeed, the proportion of the main lesser kestrel foraging habitat (arable land), which is the main land use in both study areas, was very similar between the inter-colony and the outer-colony areas both in Apulia and Sicily (Figs 4 and S1).

## Discussion

Our findings clearly showed that home ranges of lesser kestrels from neighbouring colonies were spatially segregated during the nestling-rearing period, with home ranges of birds from different colonies overlapping less than expected by chance, resulting in space partitioning. This pattern of spatial segregation was observed in two geographically distinct populations (Apulia and Sicily) and occurred both between two very large (Apulia) and three small (Sicily) neighbouring colonies. Moreover, colonies were associated to specific exploited areas (the colony “hinterland”^43^, with individuals from the same colony showing overlapping home ranges, supporting the idea that individuals belonging to the same colony share information on the location of profitable foraging grounds.

In colonial species, areas surrounding the colonies are likely to rapidly become resource-depleted (Ashmole’s halo), and increasing colony size is expected to translate into faster resource depletion and/or progressive expansion of foraging ranges in the course of the breeding season^33^. If colonies are physically close by, intraspecific competition between colonies may then arise, because individuals from different colonies may target the same foraging areas located between colonies. Due to the relatively higher density of foraging individuals, those areas may rapidly become resource-depleted. Such areas may thus become progressively avoided, possibly leading to spatial segregation of foraging areas between birds from different colonies. Segregation may result from individuals preferentially performing foraging trips directed away from any neighbouring colony. For instance, this has been clearly shown for northern gannets (*Morus bassanus*) breeding in 12 neighbouring colonies fringing the coastline of the British Isles and Northern France, whose trips towards at-sea foraging areas were directed away from closely neighbouring colonies^23^.

The avoidance of overexploited foraging areas between neighbouring colonies is a density-dependent process: spatial segregation is in fact expected to be reinforced with increasing size of neighbouring colonies, which imply a greater local density of foraging individuals in the area that is lying between colonies. Our observation of spatial segregation occurring also between birds from neighbouring small colonies may at first seem surprising, since it may be hypothesized that density-dependent spatial segregation should be detectable only between large neighbouring colonies, whereas competition between colonies should be relaxed when colony size is small^23^. This would be the case if the distance between pairs of large and small neighbouring colonies is similar and/or the area of potential overlap between home ranges of foraging individuals from different colonies is comparable in both large and small colonies. In our case, the pairwise distances between small neighbouring colonies are considerably smaller than those between large colonies, and the area of potential overlap between home ranges among small neighbouring colonies is considerably smaller than among large ones (Fig. 2). Although there may be a much smaller absolute number of individuals potentially targeting the inter-colony areas between small than between large neighbouring colonies, the density of foraging conspecifics in such inter-colony areas (number of kestrels foraging per unit area) may be similar in either case. Hence, overexploitation of areas located between colonies is likely to occur also between small colonies.

The comparison of land use of outer-colony areas with that of inter-colony areas did not reveal any differences for both Apulian and Sicilian colonies. Arable land, which is the main foraging habitat of the lesser kestrel, was the predominant land use class in all cases. Hence, we can safely rule out that the spatial segregation patterns we observed resulted from the presence of unsuitable foraging habitats in the inter-colony areas.

Despite several studies investigating the spatial ecology of colonial landbirds and mammals, such as vultures^44,45^ and bats^46,47^, to our knowledge evidence for spatial segregation of home ranges between individuals from neighbouring colonies has been lacking so far in terrestrial animals, with the single exception of the lesser kestrel^48^. Our findings support the idea that mitigation of intraspecific competition between individuals from neighbouring colonies by means of spatial segregation of exploited areas is a general pattern among colonial species.

## Methods

### Target species and study areas

European populations of the lesser kestrel breed mostly around the Mediterranean Sea in pseudo-steppe and open farmland landscapes. Lesser kestrels mostly overwinter in sub-Saharan Africa, returning to the breeding areas in February/March^35^. Between late April and early May, pairs are formed and females lay 3–5 eggs (single brooded). Incubation lasts ca. 30 days and nestlings fledge at ca. 35–40 days. After hatching, at least one pair member spends the night inside the nest until the late nestling-rearing stage, when both pair members shift to frequenting large communal night roosts.

The study was carried out in two geographically distinct populations, both in Southern Italy: one in Apulia and the other in Sicily. In Apulia, we collected data at two large urban colonies that are ca. 10 km apart, Altamura (40°49′N; 16°33′E) and Gravina in Puglia (40°49′N; 16°25′E). Altamura and Gravina in Puglia are small cities (ca. 50–70000 inhabitants) hosting large colonies of ca. 1000 breeding pairs each^48^. Both cities are surrounded by extensive pseudo-steppe farmland landscapes (mostly cereal steppe habitats) where lesser kestrels forage. In both colonies, we relied on birds nesting in nestboxes placed on the terraces of large buildings located in the old towns (see also Podofillini *et al*.^49^. In Sicily, the study was conducted in the Gela Plain (37°07′N; 14°20′E) at three small colonies (ca. 11–32 breeding pairs), which are located ca. 5 km apart (Fig. 2). Colonies are settled on rural buildings, often abandoned and partly decaying, which are surrounded by croplands mainly represented by wheat (*Triticum* spp.) and artichoke (*Cynara* spp.) alternated with grassland and other cultivations^50^. We relied on birds nesting both in nestboxes and crevices of rural buildings.

Nests were checked twice per week from 15 April to 30 July (both in Apulia and in Sicily), recording information about laying date, brood size, hatching date, hatching success and nestling survival at 20 days from hatching of the first egg (it was difficult to follow the fate of nestlings after 20 days because most left their nest to wander around, sometimes mixing with nestlings from nearby nests^49^).

### GPS deployment

All birds were captured by hand within their nestbox or nest cavity, and equipped with GPS tags during the late incubation stage, mostly a few days before hatching. The study was conducted in accordance with relevant guidelines and regulations. Specifically, captures in Apulia were carried out by Istituto Nazionale per la Protezione e la Ricerca Ambientale (ISPRA) under the authorization of Law 157/1992 [Art.4 (1) and Art. 7 (5)] and in Sicily by the University of Palermo under authorization n. 1616/2014 issued by Regione Sicilia. We equipped with GPS tags 25 lesser kestrels from 25 different nests in Apulia (2016 breeding season), and 12 individuals from 11 nests in Sicily (2015 breeding season). We deployed solar-driven, remote-downloading GPS-UHF tags (NanoFix GEO + RF, PathTrack Ltd., UK, in Apulia and customized Pica, Ecotone, PL, in Sicily) using a backpack Teflon harness^51^. Tags were programmed to record 1 GPS position every 15 min. However, tags automatically adjusted the GPS sampling rate according to the actual battery level, preserving battery power and allowing UHF data transmission to base stations that were deployed at breeding sites. The weight of tags (NanoFix GEO + RF: 4 g; Pica: 5 g; plus 1 g of Teflon harness) was always below 5% of body mass [NanoFix GEO + RF: 3.46% (0.41 s.e.m.), range 2.77–4.20%; Pica: 3.49% (0.25 s.e.m.), range 3.33–3.92%].

Data from 18 simultaneously tracked individuals breeding in Apulia (9 from Altamura and 9 from Gravina in Puglia) and from 6 simultaneously tracked individuals breeding in Sicily (two for each colony site) were available for statistical analyses (Table 1). We excluded birds with largely malfunctioning devices (that in a few cases stopped transmitting data a few days after deployment) and those that failed reproduction and did not fledge any nestling (as they were no longer tied to the colony site and started wandering far from the colony site; our unpubl. data).

### Home range determination

To identify areas exploited by tracked birds during the nestling-rearing stage, we calculated for each individual the Utilization Distribution (UD) using the fixed kernel density estimation (KDE) with reference bandwidth (href) by means of the R package adehabitatHR^52^. To this end, we selected GPS positions according to the following criteria: 1) we considered positions collected during the 29 days after hatching of the first egg (a few individuals were tracked for a shorter period because of tag failure; see Table 1); 2) we excluded all positions collected within 50 m of the nest site (to eliminate all instances when the birds were perching close to the nest); 3) we avoided the inclusion of roosting sites, used by males and by females only during the late nestling-rearing stage, considering only GPS positions recorded between 5:00–17:00 h UTC (7–19 h local time, approximately 2 hours after sunrise and 2 before sunset); this time window was identified after exploring high-frequency tracking data (1 GPS position every minute for both day- and night-time) of lesser kestrels in southern Italy (our unpubl. data).

Because a small fraction of the individuals from each colony was tracked, we can draw inferences at the colony level only if the tracked individuals are representative of the variability of space use by colony members. To assess representativeness, we investigated for each colony how the total 95% KDE area increased with sample size, performing a bootstrap analysis according to Lascelles *et al*.^40^. For each sample size (from 1 to n – 1 individuals), we plotted a random selection of individual 95% KDEs and calculated the proportion of positions from non-selected individuals that overlapped with the sum of selected individual 95% KDEs. This process was iterated 100 times and the average overlapping proportion (“inclusion”) was calculated for each sample size. Then, we fitted a non-linear regression to inclusion values (see details of fitted function in Lascelles *et al*.^40^) and the representativeness of the tracked individuals was computed as the percentage of the estimated asymptote value reached by the highest predicted inclusion value. This test was not performed for Sicilian colonies due to the small sample size (see “*GPS deployment”*). Computations were performed in R 3.3.1^53^.

In order to rule out possible sources of bias when comparing home ranges between colonies, we checked for variation in the duration of the tracking, sampling periods, and breeding success between colonies and sexes (comparisons were made within each study area, Apulia and Sicily; sex effects were not tested for Sicilian birds since only one male was tracked). There were no statistically significant differences between colonies (or sexes in Apulia) in the number of days tracked for each individual (linear models; Apulia, colony: *F*_1,15_ = 0.21, *p* = 0.66; sex: *F*_1,15_ = 0.07, *p* = 0.79; Sicily, colony: *F*_2,3_ = 0.92, *p* = 0.48), in the end date of tracking (Apulia, colony: *F*_1,15_ = 0.23, *p* = 0.64; sex: *F*_1,15_ = 1.27, *p* = 0.28; Sicily, colony: *F*_2,3_ = 3.86, *p* = 0.15) and in the number of nestlings at day 20 (Apulia, colony: *F*_1,15_ = 0.02, *p* = 0.89; sex: *F*_1,15_ = 0.39, *p* = 0.54; Sicily, colony: *F*_2,3_ = 0.20, *p* = 0.83). We could therefore rule out the possibility that systematic differences between colonies and sexes in tracking effort and breeding success biased our findings concerning the spatial distribution of home ranges.

### Statistical analysis of home range segregation

We estimated the magnitude of spatial segregation between home ranges of individuals belonging to different neighbouring colonies separately for each study population (i.e. the two neighbouring Apulian colonies and the three neighbouring Sicilian colonies) by means of a randomization procedure. We first built a home range overlap matrix between individuals belonging to both the same colony and neighbouring colonies according to the UD. The UD overlap between a pair of individuals *i*,*j* was calculated using the Utilization Distribution Overlap Index (UDOI), as recommended by Fieberg & Kochanny^41^, by means of the *kerneloverlap* function of the adehabitatHR R package^52^. The UDOI is an home range overlap index which assumes that different individuals use space independently of one another^41,42^. UDOI values range from zero (no overlap) to 1 (uniformly distributed and have 100% overlap; it can however be >1 when UDs are non-uniformly distributed and have a high degree of overlap)^41^. To compute the UDOI home range overlap matrix, we specified a grid extent equal to 1 and a grid size equal to 200 in the *kerneloverlap* function. We then built a second matrix of colony membership, whereby each pair of individuals *i*, *j* was coded as 0 if both individuals belonged to the same colony, and 1 if they belonged to different colonies. After removing diagonals from both matrices, we computed a correlation coefficient (Pearson’s *r*; *r*_obs_ hereafter) between the two matrices. Because of the coding of colony membership, highly negative values of *r*_obs_ indicate that 1) home ranges of individuals belonging to the same colony are highly overlapping, and that 2) those of individuals belonging to different colonies are deeply segregated. We then randomly and independently rotated each individual set of positions (by anchoring it to the coordinates of its own breeding site; see stars in Fig. 2) 9999 times and calculated each time a new home range overlap matrix, which was correlated with the colony membership matrix. By this way, we obtained a distribution of *r* values representing the null hypothesis of random spatial distribution of home ranges around the breeding site, assuming that individuals were free to move in the space surrounding the colonies while remaining tied to their breeding site. In the analysis of data from the three Sicilian colonies, we deleted from the overlap matrix the data referring to the two most distant colonies, which were non-neighbouring (see Fig. 2). Significance of *r*_obs_ was calculated as the probability (*p*_rand_) of obtaining a more negative value than *r*_obs_. All computations were performed in R 3.3.1^53^.

Because nestling-feeding lesser kestrels search for food in areas that can be widely scattered in the surroundings of the breeding site but frequently return to the breeding site to deliver food to their progeny, the UD had invariably higher values on the breeding site (see Supplementary Figs S2 and S3). The UD might thus overemphasize the exploitation of the areas in the immediate surroundings of the breeding site (where the birds did not forage), at the same time underestimating the importance of the use of foraging areas located away from the breeding site (see Supplementary Figs S2 and S3), which are important in an inter-colony foraging competition perspective. To assess the robustness of our conclusions, we therefore repeated the analyses by using only the GPS positions located outside the urban area of the cities where tracked birds breed in Apulia (see Fig. 2; these urban areas are not used for foraging; urban areas identified by Corine Land Cover 2012, CLC12 hereafter; https://land.copernicus.eu/pan-european/corine-land-cover/clc-2012; code 111, continuous urban habitat). Such a procedure could not be applied to data from the Sicilian colonies, where birds breed on isolated buildings in open farmland landscapes.

### Comparison of land use in the areas surrounding colonies

To rule out that spatial segregation between neighbouring colonies is actually due to uneven distribution of suitable foraging habitats in the areas surrounding colonies, we described land use in these areas. Because the inter-colony areas appeared to be avoided in both Apulia and Sicily (Fig. 2), we assessed whether the availability of pseudo-steppe and open farmland landscapes (i.e. the main lesser kestrel foraging habitat^35^) was lower in the inter-colony areas than in the rest of the areas surrounding each colony. For each pair of neighbouring colony we created a buffer area centred on the nesting site whose radius was equal to the distance between the two nesting sites. The intersection of the two buffers created three areas for each pair of neighbouring colonies: one inter-colony area and two different areas surrounding colonies (outer-colony areas), one for each colony (see Fig. 4). We then calculated the proportion of each land use type from CLC12 within each of these three areas by means of ESRI ArcMap 10.2.1 for Desktop (see Supplementary Fig. S1). We pooled together CLC12 land use categories that were similar in habitat and structure, hence obtaining 7 land use classes: urban areas (urban fabric; industrial, commercial and transport units), arable land, permanent crops (vineyards; fruit tree and berry plantations; olive groves), grasslands (pastures; natural grasslands), heterogeneous cultivations (heterogeneous agricultural areas), wooded areas (forests; scrub and/or herbaceous vegetation associations), water bodies.

### Data Accessibility

The datasets generated during and/or analysed during the current study are available from the corresponding author on reasonable request.

## Electronic supplementary material

Supplementary information
