# Supplementary material for: Spatial segregation of home ranges between neighbouring colonies in a diurnal raptor
Source: Sci Rep. 2018 Aug 6;8:11762. doi: 10.1038/s41598-018-29933-2 (PMC6078973; doi:10.1038/s41598-018-29933-2)

SUPPLEMENTARY INFORMATION

Spatial segregation of home ranges between neighbouring colonies in a diurnal raptor

Jacopo G. Cecere[1]*, Salvatore Bondì[2], Stefano Podofillini[3], Simona Imperio[1], Matteo Griggio[4], Egidio Fulco[5], Andrea Curcio[3], Delphine Ménard[3], Ugo Mellone[6], Nicola Saino[3], Lorenzo Serra[1], Maurizio Sarà[2], Diego Rubolini[3]*

1. *Area Avifauna Migratrice, Istituto Superiore per la Protezione e la Ricerca Ambientale (ISPRA), via Ca' Fornacetta 9, I-40064 Ozzano Emilia (BO), Italy*
2. *Laboratorio di Zoogeografia ed Ecologia Animale (LABZEA), Dipartimento STEBICEF, Università degli Studi di Palermo, Via Archirafi 18, I-90123 Palermo, Italy*
3. *Dipartimento di Scienze e Politiche Ambientali, Università degli Studi di Milano, via Celoria 26, I-20133 Milano, Italy*
4. *Dipartimento di Biologia, Università degli Studi di Padova, via U. Bassi 58/B, I-35131 Padova, Italy.*
5. *Studio Naturalistico Milvus, via F.lli Perito snc, I-85010 Pignola (PZ), Italy*
6. *Vertebrates Zoology Research Group, Departamento de Ciencias Ambientales y Recursos Naturales, University of Alicante, Apdo. 99, Alicante E-03080, Spain*

*Correspondence: Jacopo G. Cecere (jacopo.cecere@isprambiente.it; ORCID: 0000-0002-4925-2730), Diego Rubolini (diego.rubolini@unimi.it; ORCID: 0000-0003-2703-5783)

Figure S1. Land use map (according to land use classes defined in the Methods and Corine Land Cover 2012) of outer- (coloured buffers) and inter-colonies areas (grey areas). Top panel: Apulian colonies, red line = Gravina in Puglia (GRA), blu line = Altamura (ALT). Low panel: Sicilian colonies, red line = Canalotto (CAN), green line = San Gregorio (SAN), blue line = Torrevecchia (TOR). Images elaborated with ESRI ArcGIS 10.2.1. for Desktop.

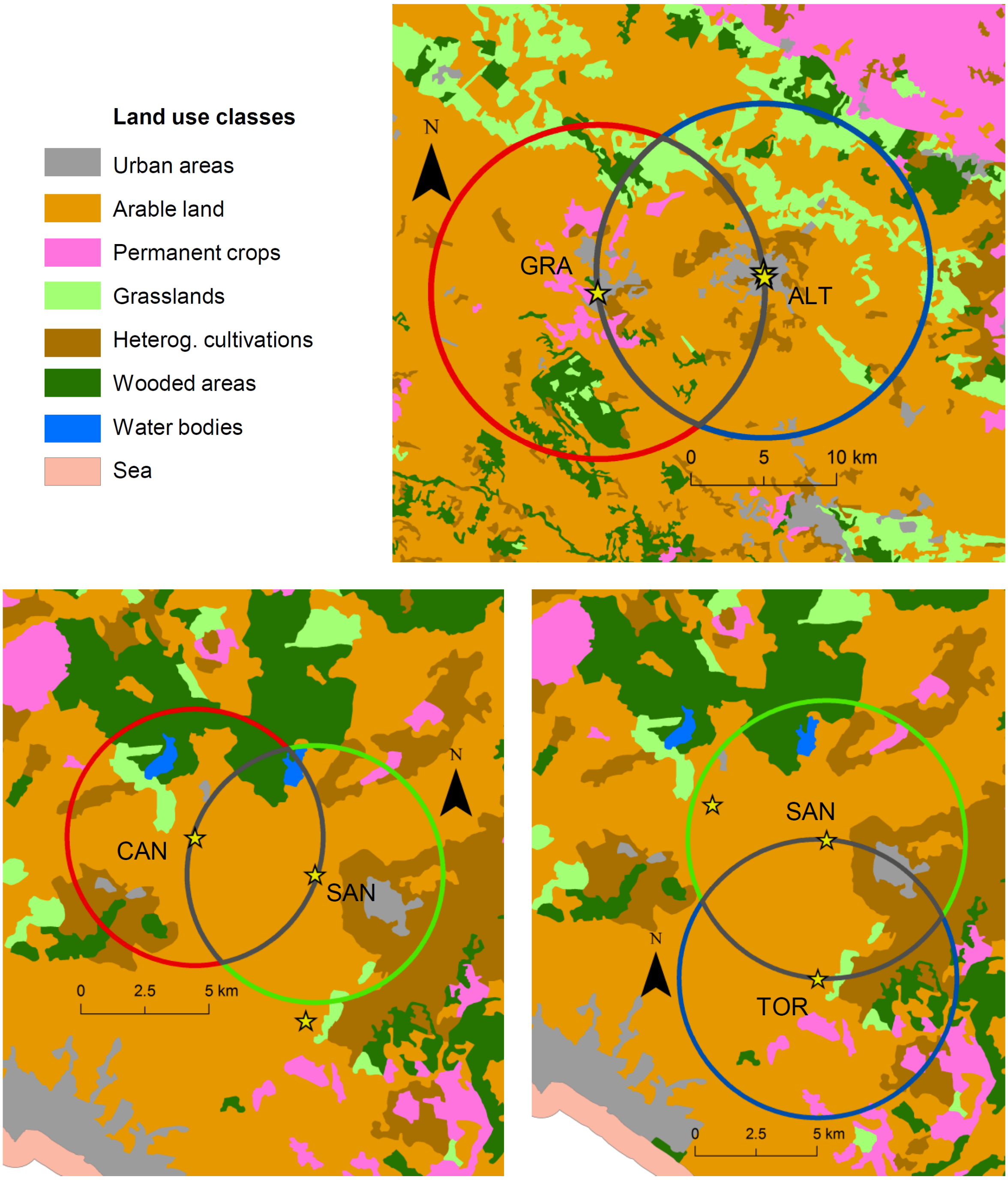

Figure S2. Maps of selected GPS positions (left panels) and volume UD (with 95% KDE) (right panels) for each tracked individual in Apulia. The position of the breeding sites are marked with yellow triangles. Individuals (see list in Table 1) are sorted according to colony site (GRA = Gravina in Puglia, red dots; ALT = Altamura, blue dots). The corresponding metric unit is shown in the first map.

Map data: Google, TerraMetrics 2018, plotted with the R package *ggmap* (Kahle & Wickham 2013).

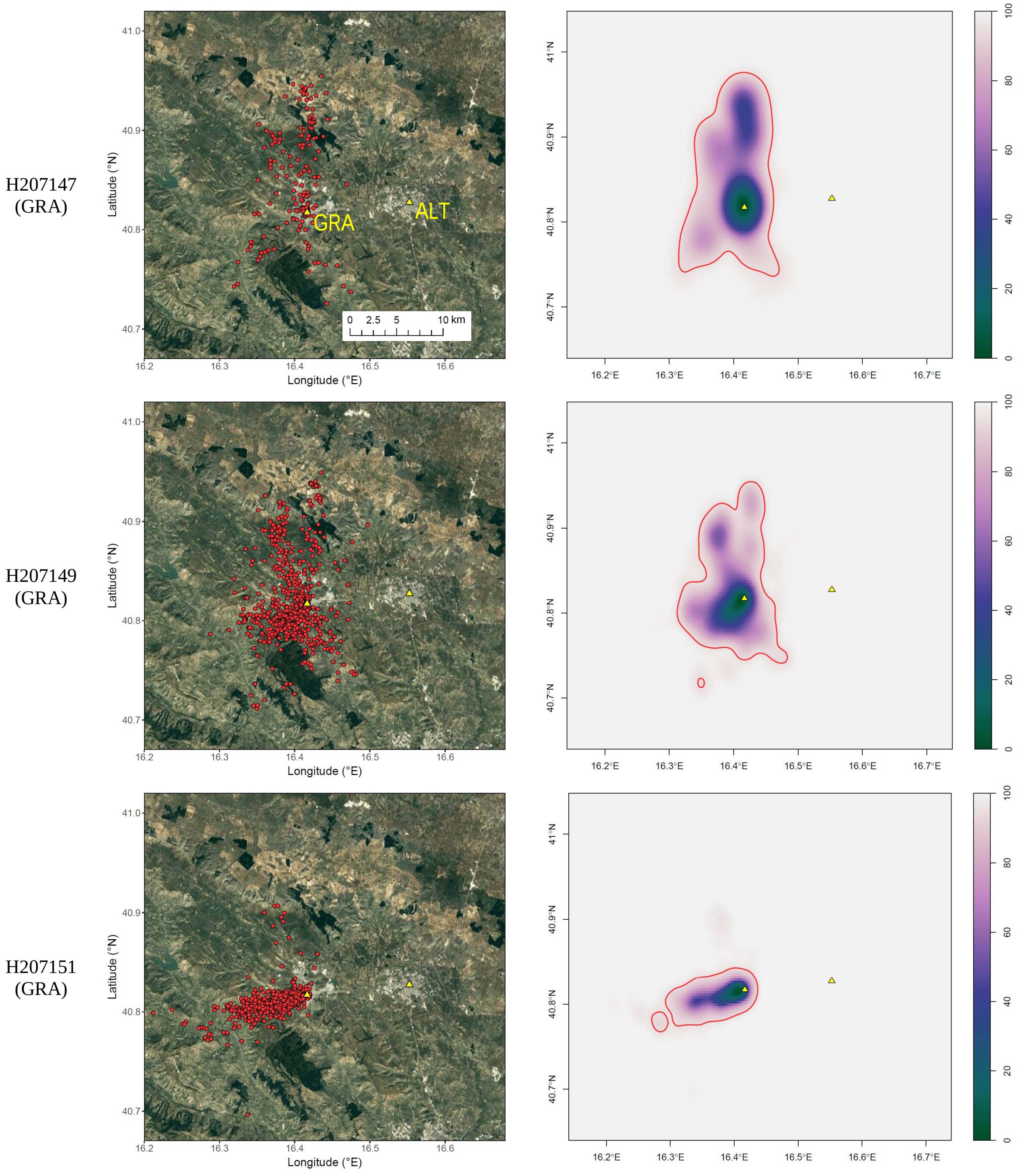

Kahle D. & Wickham H. ggmap: Spatial Visualization with ggplot2. *The R Journal*. **5**, 144–161 (2013).

Figure S2. Continued

H207154
(GRA)

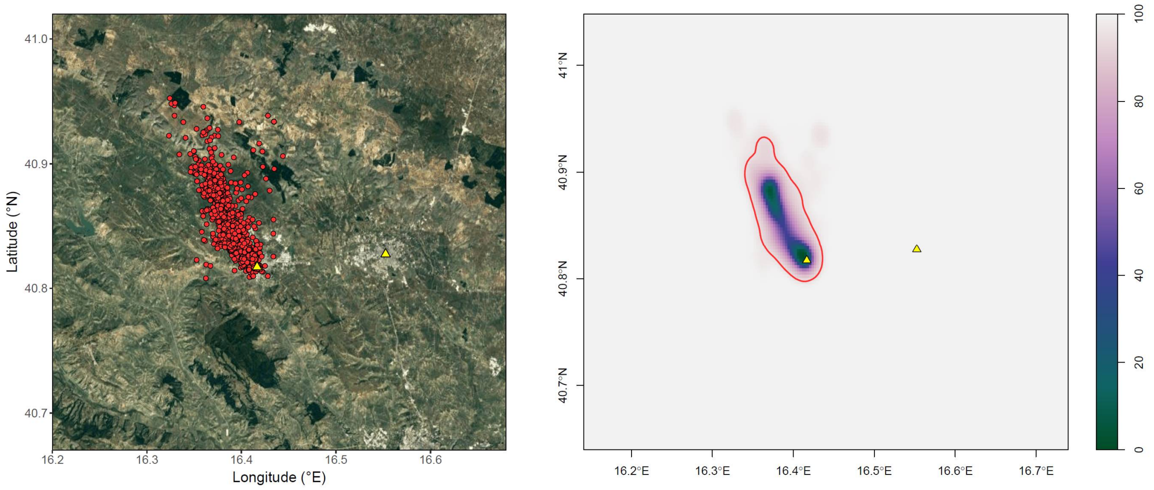

H207155
(GRA)

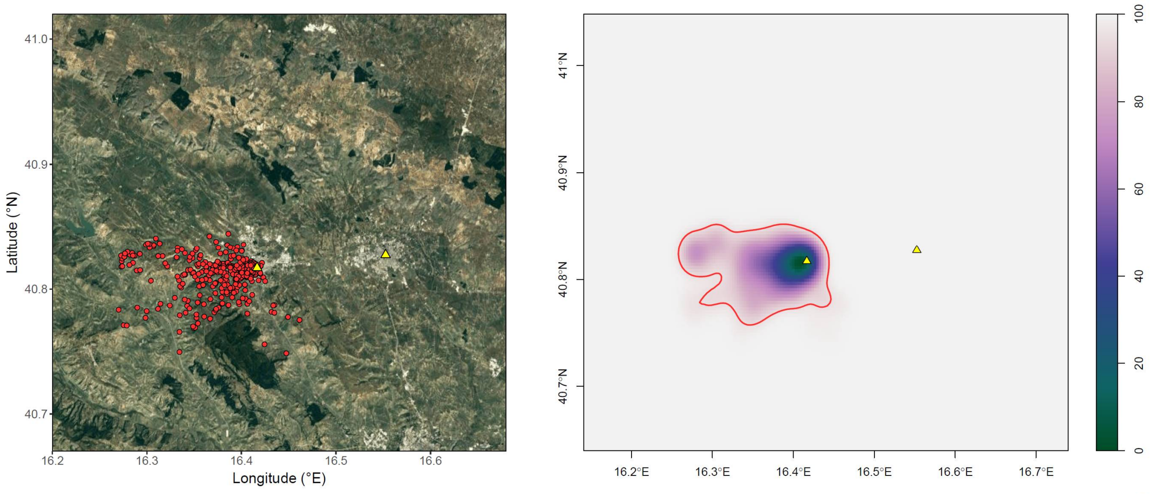

H207156
(GRA)

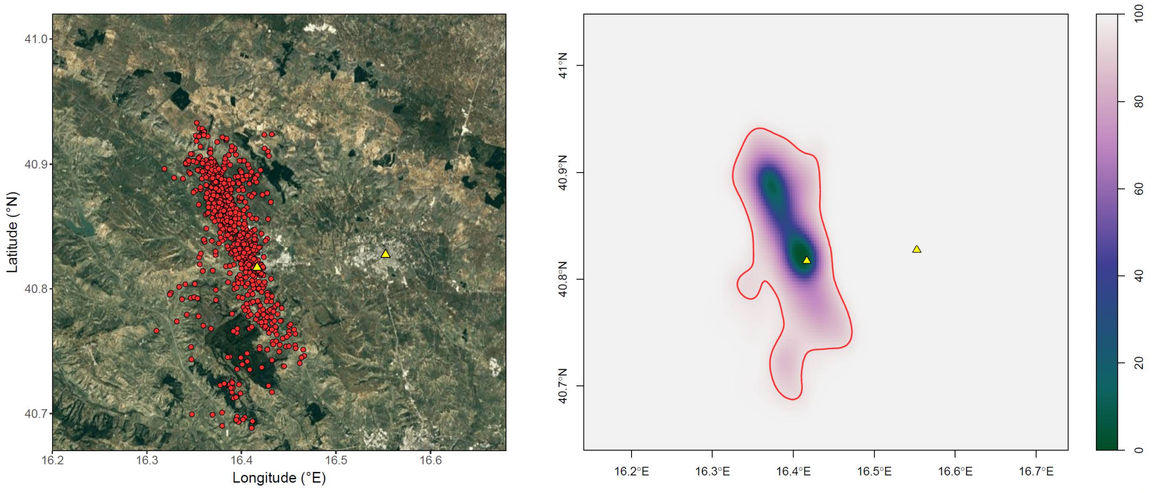

H207200
(GRA)

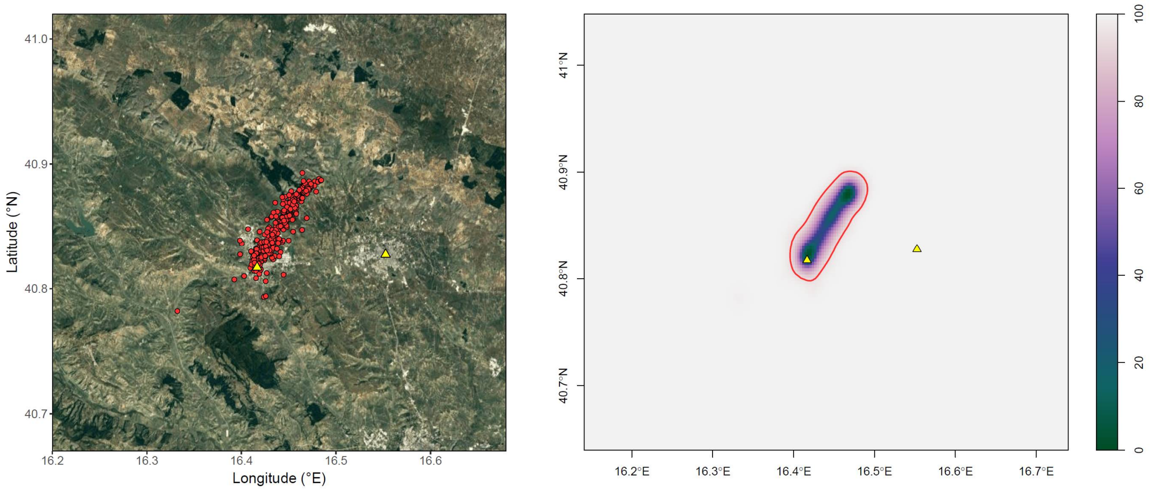

Figure S2. Continued

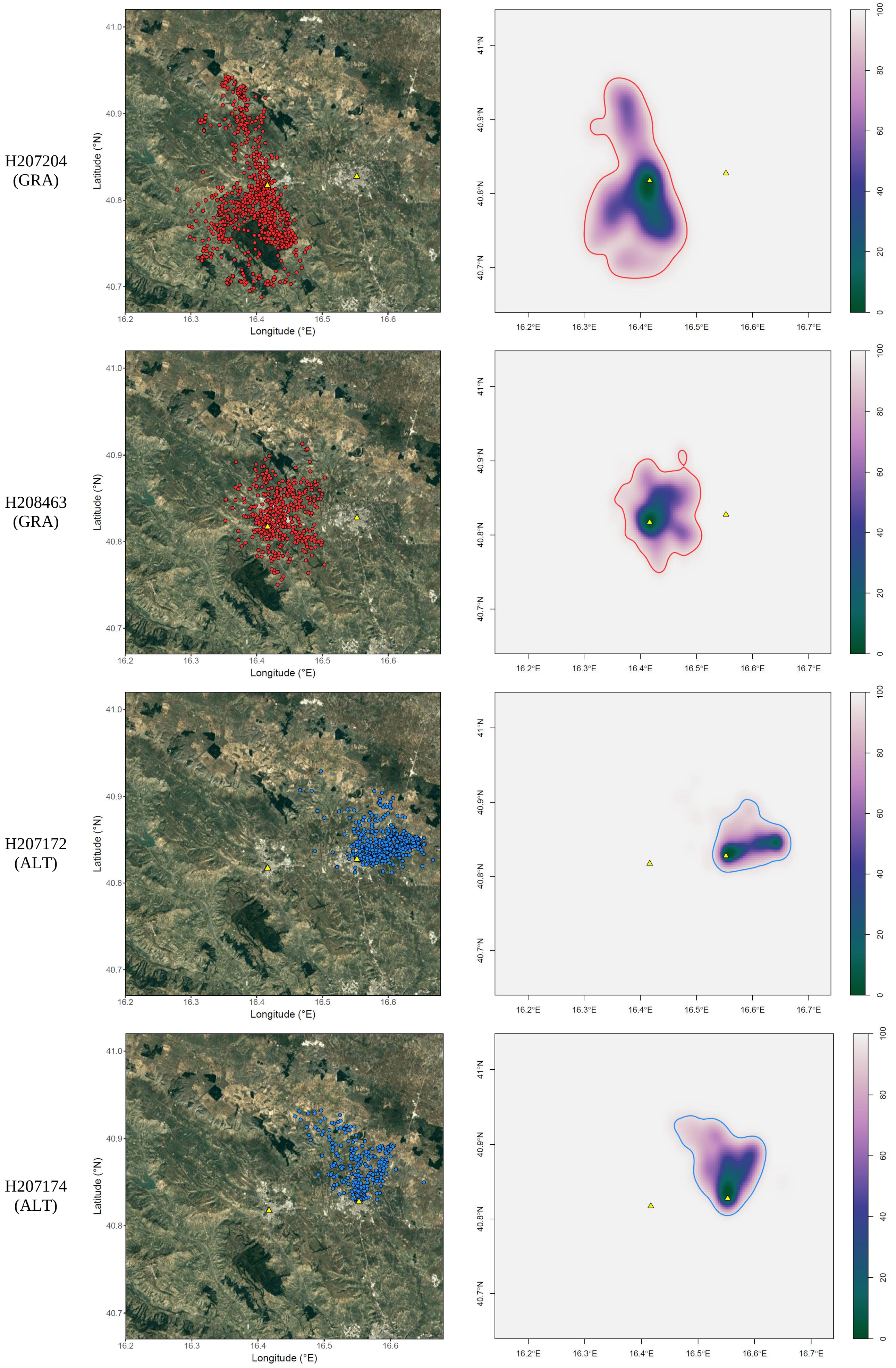

Figure S2. Continued

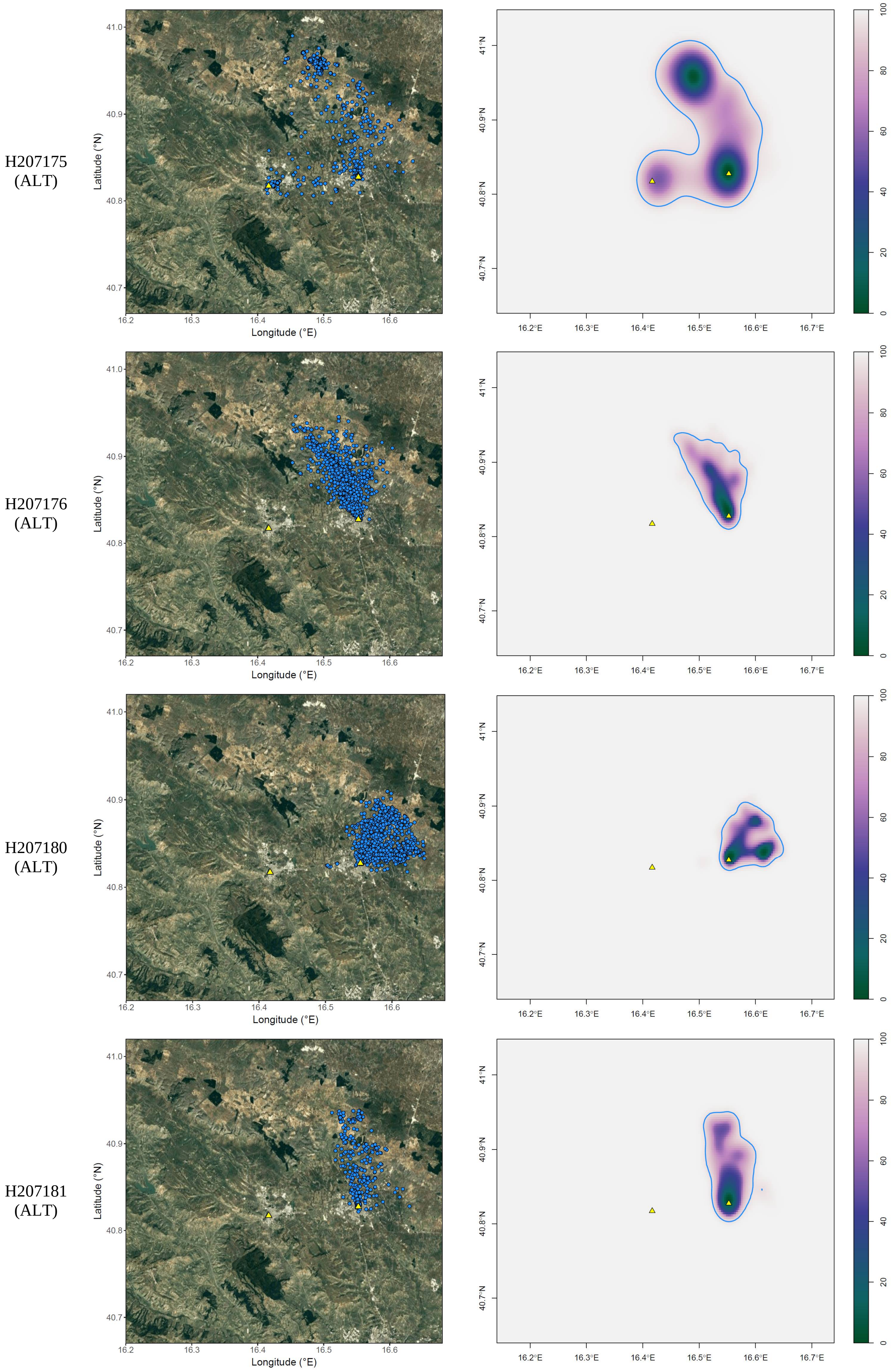

Figure S2. Continued

H207216
(ALT)

H207220
(ALT)

H207222
(ALT)

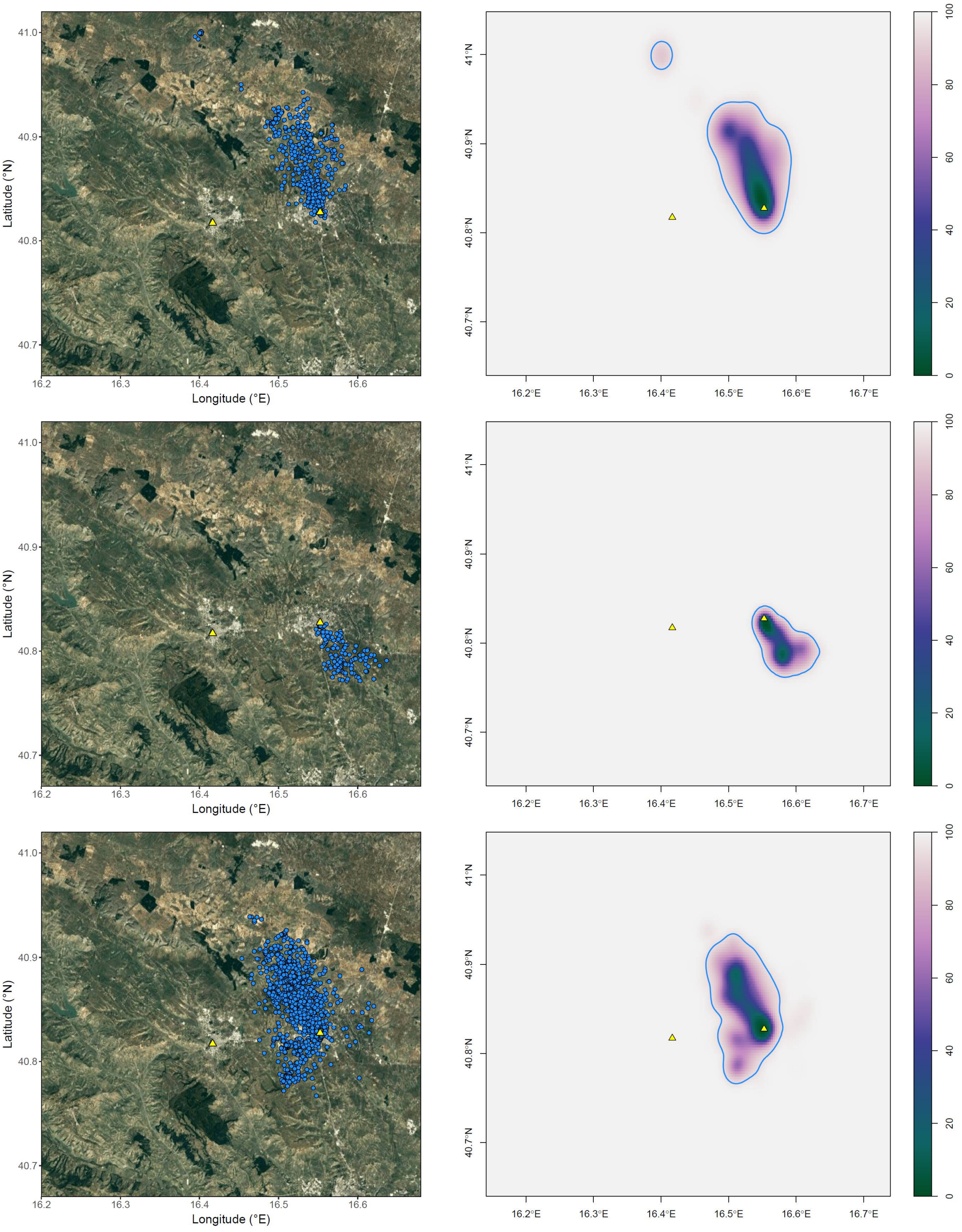

Figure S3. Maps of selected GPS positions (left panels) and volume UD (with 95% KDE) (right panels) for each tracked individual in Sicily. The position of the breeding sites are marked with yellow triangles. Individuals (see list in Table 1) are sorted according to colony site (CAN = Canalotto, blue dots; SAN = San Gregorio, green dots; TOR = Torrevecchia, red dots). The corresponding metric unit is shown in the first map.

Map data: Google, TerraMetrics 2018, plotted with the R package *ggmap* (Kahle & Wickham 2013).

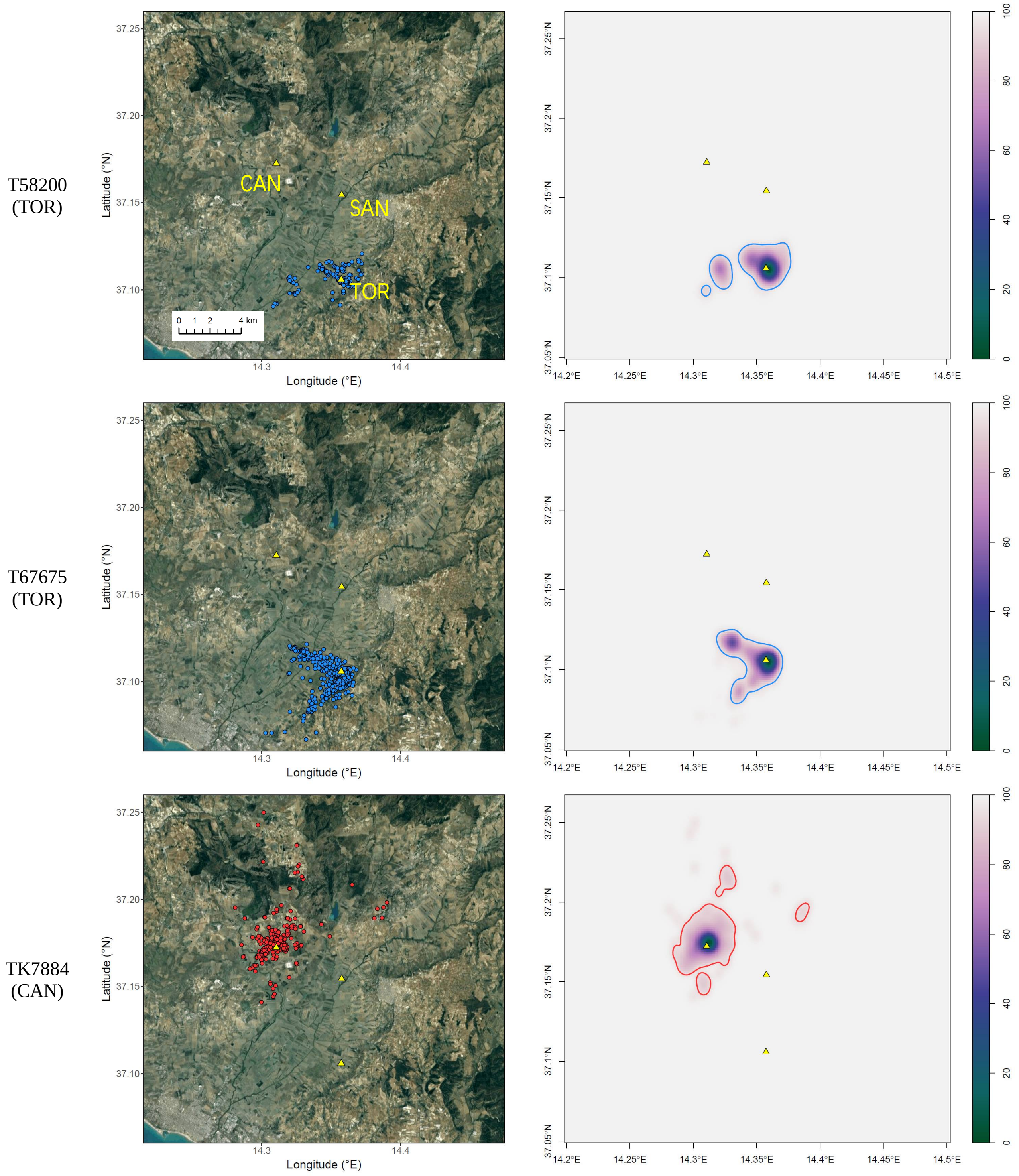

Kahle D. & Wickham H. ggmap: Spatial Visualization with ggplot2. *The R Journal*. **5**, 144–161 (2013).

Figure S3. Continued

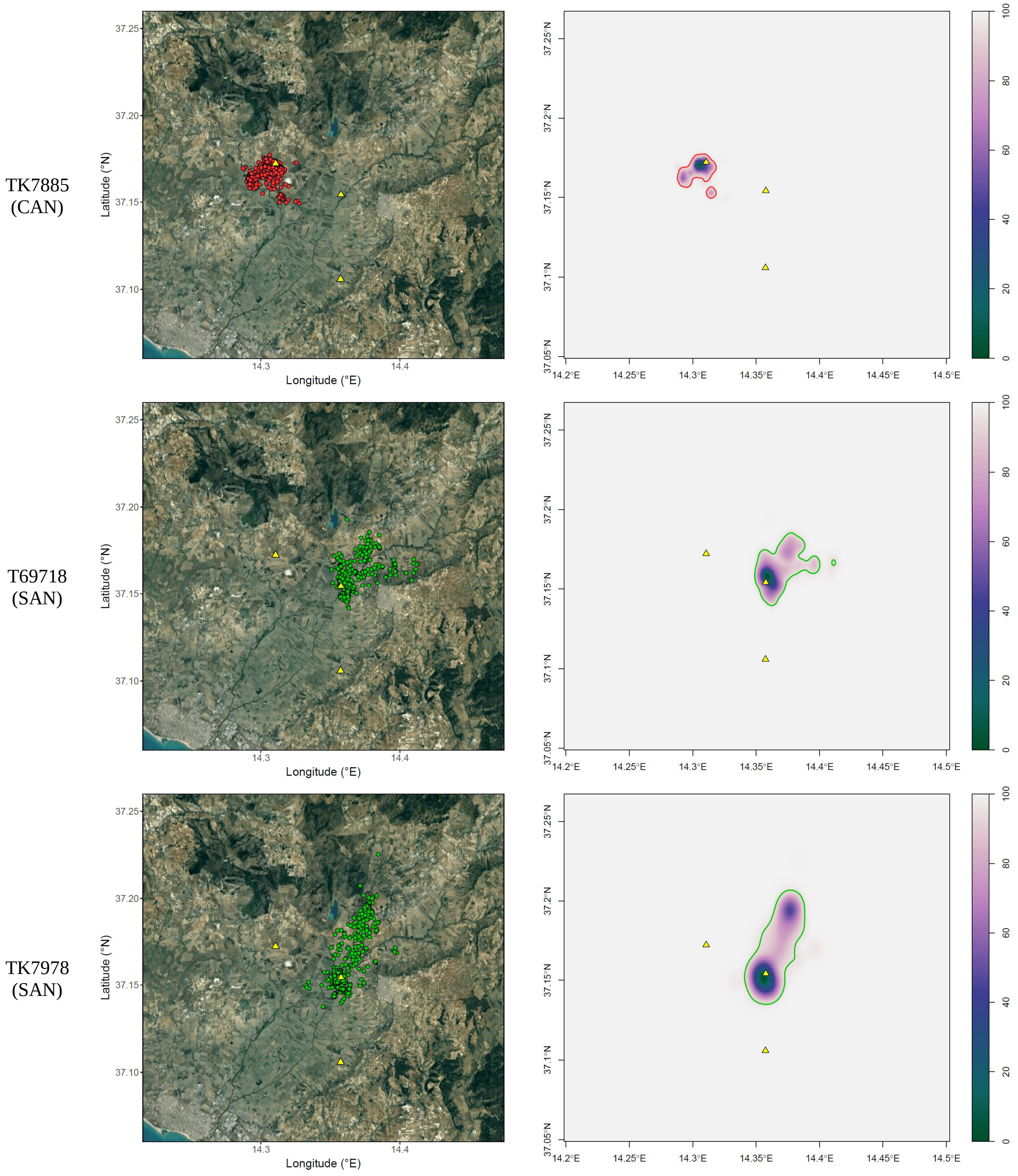

Supplement: Supplementary file 1 — Supplementary information [file 41598_2018_29933_MOESM1_ESM.pdf]
